# Patients near death receiving specialized palliative home care being transferred to inpatient care – a registry study

**DOI:** 10.1186/s12904-024-01549-6

**Published:** 2024-08-24

**Authors:** Camilla Wall, Karin Blomberg, Elisabeth Bergdahl, Helena Sjölin, Fredrik Alm

**Affiliations:** 1 Department of Oncology, Faculty of Medicine & Health, Örebro University, SE 70182, Örebro, Sweden; 2 School of Health Sciences, Faculty of Medicine & Health, Örebro University, SE 70182, Örebro, Sweden

**Keywords:** Palliative care, Palliative medicine, Transfers of patients

## Abstract

**Background:**

The majority of palliative care patients express a preference for remaining at home for as long as possible. Despite progression of disease there is a strong desire to die at home. Nonetheless, there are transfers between care settings, demonstrating a discrepancy between desired and actual place of death.

**Aim:**

To map the prevalence of patients near death undergoing specialized palliative home care and being transferred to inpatient care in Sweden.

**Methods:**

A national retrospective cross-sectional study based on data from the Swedish Register of Palliative Care. Patients ≥ 18 years of age enrolled in specialized palliative home care with dates of death between 1 November 2015 and 31 October 2022 were included (*n* = 39,698). Descriptive statistics were used.

**Results:**

Seven thousand three hundred eighty-three patients (18.6%), approximately 1,000 per year, were transferred to inpatient care and died within seven days of arrival. A considerable proportion of these patients died within two days after admission. The majority (73.6%) were admitted to specialized palliative inpatient care units, 22.9% to non-specialized palliative inpatient care units and 3.5% to additional care units. Transferred patients had more frequent dyspnoea (30.9% vs. 23.2%, *p* < 0.001), anxiety (60.2% vs. 56.5%, *p* < 0.001) and presence of several simultaneous symptoms was significantly more common (27.0% vs. 24.8%, *p*  0.001).

**Conclusion:**

The results show that patients admitted to specialized palliative home care in Sweden are being transferred to inpatient care near death. A notable proportion of these patients dies within two days of admission. Common features, such as symptoms and symptom burden, can be observed in the patients transferred. The study highlights a phenomenon that may be experienced by patients, relatives and healthcare personnel as a significant event in a vulnerable situation. A deeper understanding of the underlying causes of these transfers is required to ascertain whether they are compatible with good palliative care and a dignified death.

## Introduction

Globally, people are living longer than ever before. According to the World Population Prospects 2022, the number of people aged 60 years and over currently exceeds the number of children under five years [1]. Consequently, the proportion of people with palliative care needs will increase in the near future [1, 2]. As defined by the World Health Organisation (WHO), palliative care is an approach that aims to improve the quality of life of patients and their families facing life-threatening illness. This is achieved by preventing and relieving suffering through the early identification, assessment and treatment of pain and other physical, psychosocial and spiritual problems [3]. Concurrently, the International Association for Hospice and Palliative Care (IAHPC) characterises palliative care as an approach that considers the needs of all individuals afflicted with a serious illness, particularly at the end of life [4]. It is thereby evident that these definitions diverge to a slight extent from one another. In Sweden specialized palliative care refers to care performed by a multi-professional team with special competence and knowledge. This type of care is offered to patients with complex symptoms or with a life situation that entails special needs [5, 6]. Specialized palliative care can be performed in hospitals or other institutions and at home. When patients are cared for at home, their palliative care needs should be met with the help of general or specialized home care [5, 6]. Specialized palliative care requires health care available around the clock, every day of the week. The patient should at least have access to in-person home visits by a nurse and authorized personnel access to a palliative medicine hotline [7].

In Sweden and many other parts of the world, the majority of palliative care patients express a preference for remaining at home for as long as possible [8, 9]. Despite the progression of their disease and the associated worsening of symptoms, there is a strong desire to die at home [8–10]. Nonetheless, there are cases of transfers between care settings, which demonstrate a discrepancy between the desired place of death and the actual place of death [11–14]. Prior research has indicated that the probability of transfer is elevated during the final three months of life, with an increased risk as death draws near [12, 15]. A higher incidence of hospitalisation in the final week of life is observed in patients receiving care at home compared to those residing in nursing homes [15]. In certain circumstances, the necessity and justification of these transfers may be perceived even in the context of a patient who is close to death [11, 13, 15]. For instance, in situations where the patient’s condition requires care that can only be provided in an inpatient setting [11]. In other circumstances, transfers near death may be perceived as potentially avoidable. In this context, the term “potentially avoidable” refers to those conditions that are considered to be safely and effectively manageable even outside an acute hospital setting [11, 16]. Regardless of the rationale behind the transfer, it is crucial to strive to minimise the impact of the transfer process on patients, families, and healthcare personnel across different settings [13]. To the best of our knowledge, no previous studies have investigated the prevalence of transfers of patients near death (< 7 days) in Sweden. The objective of this study was therefore to map the prevalence of patients near death undergoing SPHC and being transferred to inpatient care facilities.

## Material and methods

### Study design

This study is a national retrospective cross-sectional study based on data from the Swedish Register of Palliative Care (SRPC).

### Participants

All patients registered in the SRPC between 1 November 2015 and 31 October 2022, aged 18 years or older who received SPHC were included in the study.

### Setting

The SRPC is a nationwide quality register established in 2005 and includes data on all types of deaths, both expected and unexpected, in Sweden (a country with 10-plus million inhabitants). The registry is well established, and except for two municipalities not enrolled, all county councils and all municipalities in Sweden report to the SRPC. The registry has about 55–60% coverage of all deaths (mapped against the Swedish Tax Agency/Population Registry). Healthcare personnel at the unit where the death occurs, in most cases nurses, register the patient’s death using a questionnaire filled in directly via the register’s website [17]. The questionnaire is developed by the SRPC and currently consists of 27 questions about end-of-life care, including gender, age, date of death, hospitalisation, place of death, diagnosis, symptoms, symptom management and palliative care decisions [18]. The questions are answered retrospectively based on data extracted from medical record, with reference to the patient’s last week of life. It should be noted that prior to 2020, data on unexpected deaths were not collected in detail. Following the year 2020, all questions on the questionnaire must be answered, regardless of whether the death is classified as expected or unexpected. The SRPC also collects data from a questionnaire that relatives of the deceased answer; data not used in this study [19].

### Variables

The outcome was whether patients enrolled in specialized palliative home care (SPHC) were transferred from home to inpatient care and died within 7 days of enrolment. In this study, the term “transfer” is defined as the movement of patients from their place of residence, where they receive SPHC, to some form of inpatient care.

All data are registered in the SRPC using fixed checkbox options. Two variables were in focus: “enrolled from”, with an associated transfer date, and “place of death”, with the associated date of death. Both variables were reported to the SRPC using the following checkbox options: special accommodation/nursing and care accommodation/LSS (LSS = accommodation that is included under the “Act on support and services for certain disabled persons”); short-term care facility; non-specialized palliative inpatient hospital care (ward/reception/ICU); specialized palliative inpatient hospital care; own home with the support of specialized palliative home care; own home with the support of general home health care; own home with the support of home care; own home without support; or other. In addition, the following data were retrieved from the SRPC: sex, age, diagnosis, symptoms.

First, the study population was restricted to patients enrolled in SPHC, by selecting only patients with the place of death recorded as SPHC or patients transferred from SPHC with the place of death recorded as inpatient care unit or other care unit (special accommodation/nursing and care accommodation/LSS, short-term care facility/other).

Second, this population was divided into two groups of patients: (1) Patients transferred and admitted to inpatient care with death within seven days of arrival (transferred group), (2) Patients not transferred and admitted to inpatient care, or transferred and admitted longer than seven days before the time of death (other group).

### Quantitative variables

To analyse the complexity of symptoms and symptom burden, related symptoms were categorized together, resulting in the following categories of symptoms: confusion/anxiety, pain/severe pain, dyspnoea/respiratory secretion, and nausea. By related symptoms, we mean symptoms that may subjectively express themselves in a similar way. Registered responses of “don’t know” and “not answered” are reported as missing values.

### Statistical methods

Continuous variables are presented as mean with standard deviation (SD) and/or median with minimum and maximum (range). Categorical variables are presented as numbers (n) and percentages (%). For two-group comparison, Chi Square test has been used on categorical/nominal data and independent *t*-test on continuous data. All tests were two-sided, and *p*-value < 0.01 was considered significant. The statistical analyses were performed using IBM SPSS Statistics version 28.0.0.0 [20].

## Results

### Participants

A total of 39,698 patients who received SPHC were reported to the SRPC between 1 November 2015 and 31 October 2022. Of these, 7,383 (18.6%) were transferred and admitted to inpatient care near the time of their deaths (< 7 days), and 32,315 were not transferred, or transferred and admitted longer than seven days before the time of death. General characteristics of the patients in the transferred group and patients in the other group are given in Table 1.

**Table 1:** Characteristics of the study population and comparison between transferred group and other group

	Missing n (%)	Transferred group (≤ 7 days) *n* = 7,383	Other group *n* = 32,315	*p*- value
**Sex n (%)**				0.016^§^
Men		3,979 (53.9)	16,916 (52.3)	
Female		3,404 (46.1)	15,399 (47.7)	
**Age**				
Mean (SD)		72.1 (12.1)	73.0 (12.3)	< 0.001^†^
Median (Min.–Max.)		74.0 (18–103)	74.0 (18–108)	
**Diagnosis**^**a**^** n (%)**				
Cancer		6,585 (89.2)	28,603 (88.5)	0.098^§^
Cardiovascular disease		711 (9.6)	2,961 (9.2)	0.211^§^
Lung disease		455 (6.2)	1,604 (5.0)	< 0.001^§^
Dementia		49 (0.7)	399 (1.2)	< 0.001^§^
Stroke		96 (1.3)	340 (1.1)	0.065^§^
Other neurological disease		117 (1.6)	742 (2.3)	< 0.001^§^
Diabetes		121 (1.6)	549 (1.7)	0.718^§^
Fracture		38 (0.5)	82 (0.3)	< 0.001^§^
Multimorbidity		449 (6.1)	1,732 (5.4)	0.014^§^
Infection		221 (3.0)	409 (1.3)	< 0.001^§^
Other underlying disease		210 (2.8)	682 (2.1)	< 0.001^§^
**Diagnostic burden n (%)**				< 0.001^§^
0–1 diagnosis		6,167 (83.5)	28,166 (87.2)	
> 1 diagnosis		1,216 (16.5)	4,149 (12.8)	
**Symptoms**^**b**^** n (%)**				
Pain	774 (< 0.1)	5,650 (76.5)	24,587 (76.1)	< 0.001^§^
Severe pain	7,457 (0.2)	2,019 (27.3)	8,571 (26.5)	< 0.001^§^
Respiratory secretion	77 (< 0.1)	3,271 (44.3)	15,126 (46.8)	< 0.001^§^
Nausea	1,822 (< 0.1)	1,389 (18.8)	6,314 (19.5)	< 0.001^§^
Anxiety	2,048 (< 0.1)	4,446 (60.2)	18,260 (56.5)	< 0.001^§^
Dyspnoea	1,265 (< 0.1)	2,279 (30.9)	7,509 (23.2)	< 0.001^§^
Confusion	2,441 (< 0.1)	1,715 (23.2)	8,011 (24.8)	< 0.001^§^

^§^Chi square

^†^t-test

^a^The same patient may have more than one diagnosis registered

^b^The same patient may have more than one symptom registered

### Transfers to inpatient care

**Fig. 1:**
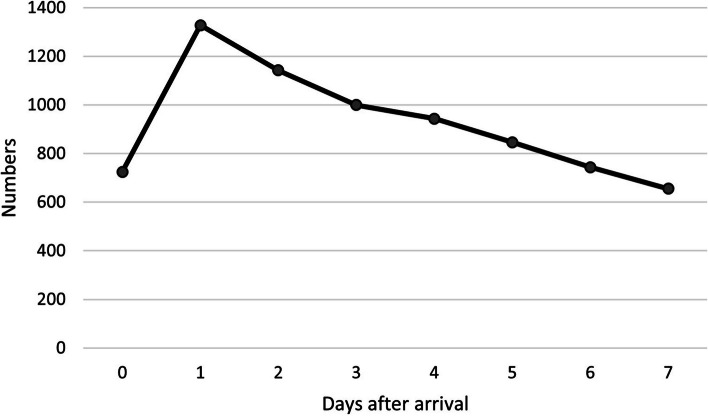
In the transferred group the number of deaths per day after arrival at the hospital

The 7,383 patients transferred to inpatient care were distributed to approximately 1000 transfers per year. Most of these patients died on day one or two after arrival (Fig. 1). The majority (73.6%) of the transferred patients were admitted to a specialized palliative inpatient care unit, 22.9% to a non-specialized palliative inpatient care unit and 3.5% to other care units (special accommodation/nursing and care accommodation/LSS; short-term care facility; other). Multiple diagnoses and diagnoses such as cardiovascular disease, stroke, fracture, infection and respiratory symptoms were more prevalent in the group admitted to non-specialized palliative inpatient care units compared to patients admitted to specialized palliative inpatient care units (Table 2).

**Table 2:** Comparison of characteristics between patients in the transferred group who were admitted to a specialized palliative inpatient care unit and those admitted to a non-specialized palliative inpatient care unit

	Missing n (%)	Hospital: Non-specialized palliative inpatient care *n* = 1,689	Hospital: Specialized palliative inpatient care *n* = 5,437	*p*-value
**Sex n (%)**				0.966^§^
Men		910 (53.9)	2,952 (53.9)	
Female		779 (46.1)	2,521 (46.1)	
**Age**				
Mean (SD)		71.7 (12.8)	72.2 (11.9)	0.158^†^
Median (Min.–Max.)		73.0 (19–102)	74.0 (18–103)	
**Diagnosis**^**a**^** n (%)**				
Cancer		1,354 (80.2)	5,034 (92.0)	< 0.001^§^
Cardiovascular disease		221 (13.1)	470 (8.6)	< 0.001^§^
Lung disease		151 (8.9)	289 (5.3)	< 0.001^§^
Dementia		14 (0.8)	33 (0.6)	0.315^§^
Stroke		56 (3.3)	38 (0.7)	< 0.001^§^
Other neurological disease		28 (1.7)	84 (1.5)	0.722
Diabetes		31 (1.8)	87 (1.6)	0.488
Fracture		21 (1.2)	16 (0.3)	< 0.001^§^
Multimorbidity		230 (13.6)	204 (3.7)	< 0.001^§^
Infection		139 (8.2)	78 (1.4)	< 0.001^§^
Other underlying disease		104 (6.2)	103 (1.9)	< 0.001^§^
**Diagnostic burden n (%)**				< 0.001^§^
0–1 diagnosis		1,233 (73.0)	4,748 (86.8)	
> 1 diagnosis		456 (27.0)	725 (13.2)	
**Symptoms**^**b**^** n (%)**				
Pain	274 (< 0.1)	1,183 (70.0)	4,305 (78.7)	< 0.001^§^
Severe pain	1,756 (0.2)	414 (24.5)	1,549 (28.3)	< 0.001^§^
Respiratory secretion	236 (< 0.1)	803 (47.5)	2,370 (43.3)	< 0.001^§^
Nausea	608 (< 0.1)	295 (17.5)	1,050 (19.2)	< 0.001^§^
Anxiety	556 (< 0.1)	882 (52.2)	3,438 (62.8)	< 0.001^§^
Dyspnoea	356 (< 0.1)	639 (37.8)	1,584 (28.9)	< 0.001^§^
Confusion	690 (0.1)	376 (22.3)	1,286 (23.5)	< 0.001^§^

^§^Chi square

^†^t-test

^a^The same patient may have more than one diagnosis registered

^b^The same patient may have more than one symptom registered

### Difference between the transferred group and the other group

No significant difference between the sexes emerged when comparing patients in the transferred group and the other group, whereas some differences were noted regarding age and diagnosis. Compared to patients in the other group, the transferred ones more often had multiple diagnoses (> 1 diagnosis, 16.5% vs. 12.8%, *p* < 0.001) and exhibited symptoms such as dyspnoea (30.9% vs. 23.2%, *p* < 0.001) and anxiety (60.2% vs. 56.5%, *p* < 0.001).

When analysing the occurrence of multiple/simultaneous symptoms (symptom burden), significantly more patients in the transferred group showed symptoms such as confusion and/or anxiety combined with pain and/or severe pain (19.0% vs 16.9%, *p* < 0.001). The proportion of patients who experienced all symptoms, apart from nausea, was also higher in the transferred group (27.0% vs 24.8%, *p* 0.001); see Table 3.

**Table 3:** Comparison of symptom burden between patients in the transferred group and the other group

	Transferred group *n* = 4,948^a^	Other group *n* = 24,401^a^	*p*-value
	n (%)	n (%)	
Confusion/anxiety	127 (2.6)	770 (3.2)	0.028
Pain/severe pain	398 (8.0)	2,192 (9.0)	0.034
Nausea	33 (0.7)	173 (0.7)	0.747
Dyspnoea/respiratory secretion	233 (4.7)	1,213 (5.0)	0.437
Confusion/anxiety + Pain/severe pain	938 (19.0)	4,133 (16.9)	< 0.001
Confusion/anxiety + Nausea	29 (0.6)	133 (0.5)	0.722
Confusion/anxiety + Dyspnoea/respiratory secretion	306 (6.2)	1,450 (5.9)	0.513
Confusion/anxiety + Pain/severe pain + Nausea	253 (5.1)	1,162 (4.8)	0.293
Pain/severe pain + nausea	123 (2.5)	654 (2.7)	0.437
Pain/severe pain + Dyspnoea/respiratory secretion	414 (8.4)	2,354 (9.6)	0.005
Pain/severe pain + Nausea + Dyspnoea/respiratory secretion	78 (1.6)	546 (2.2)	0.003
Nausea + Dyspnoea/respiratory secretion	29 (0.6)	137 (0.6)	0.833
Nausea + Dyspnoea/respiratory secretion + Confusion/anxiety	42 (0.8)	218 (0.9)	0.760
Confusion/anxiety + Pain/severe pain + Dyspnoea/respiratory secretion	1,336 (27.0)	6,054 (24.8)	0.001
Confusion/anxiety + Pain/severe pain + Nausea + Dyspnoea/respiratory secretion	386 (7.8)	1,805 (7.4)	0.324
No to all symptoms	223 (4.5)	1,407 (5.8)	< 0.001

Chi-square

^a^Patients with one or more missing values were excluded, for numbers of missing values, see Table 1

## Discussion

This national retrospective cross-sectional study, based on data from the SRPC, shows that certain patients near death enrolled in SPHC are transferred to inpatient care prior to death in Sweden.

During the years studied, approximately 1,000 transfers were made annually within the transferred group. The transfers we found represent a minimum number and may be the tip of an iceberg. The SRPC does not have full coverage and does not capture patients who died during ambulance transfer to inpatient care or those who died in the emergency department, as these patients are rarely reported to the registry. Nor does the registry capture the patients who are transferred between different inpatient care units in several stages before they die, as the registry usually only follows the most recent transfer.

Although this study was conducted in Sweden and in a SPHC context, there is reason to believe that this type of transfers also occurs in other countries and other palliative care contexts. Pivodic et al. [15] conducted a population-based study in four European countries (Belgium, the Netherlands, Italy and Spain) which included data on adult patients with expected deaths where end-of-life care had been a realistic option. The results showed that about half of the patients had been admitted to hospital at least once in the last three months of their lives. During the last seven days of life, 12–18% of patients were hospitalised [15]. This prevalence is consistent with the results of our study, although the context is different. Larsdotter et al. [21] found that the younger population who received specialized palliative care or had a palliative diagnosis at the time of death had a significantly increased risk of dying in hospital rather than at home. This is consistent with the findings of Pivodic et al. [15] showing that patients who were cared for at home were more likely to be hospitalised in the last week of life than those who were cared for in nursing homes. Through a scoping review that included 39 studies, Wilson, Birch [12] found that transitions in end-of-life care settings are common in the last year of life. This is even though most of the included studies did not quantify the number and type of transitions. Nevertheless, the results show that the risk of transitions increases as death approaches, probably as a result of arising, increasing or changing end-of-life care needs [12]. Even in the last 30 days of life, hospitalisations are common. This was found by Singh et al. [22], who included registry data from deceased patients with non-curable cancer. They found that 71% of patients were hospitalised in the last month of life. Conversely, international studies have demonstrated that transfers of patients with palliative care needs can also occur in the opposite direction, from acute care to community-based care [11, 14].

A further key finding in our study was the discovery that a considerable proportion of transferred patients died within the first two days of admission to inpatient care. Christ et al. [23] show a similar finding as their study examined the prevalence of cancer patients dying within 72 h of admission to an acute palliative care unit. One in five of the patients included spent less than three days in the unit before dying. One conclusion is that late admissions are not uncommon [23]. These findings raise the question of the potential burden that these transfers and admissions may place on patients nearing the end of their lives. A scoping review by Hanna et al. [24] has identified that burdensome transitions can be identified if any of the following criteria are met: “(1) three or more transitions to acute care facilities (Hospital, ICU, ED) in the last 90 days of life, (2) two or more transitions to acute care facilities or one or more admission to ICU in the last 30 days of life, (3) one or more transitions to acute care facilities or hospice in the last three days of life or (4) any transitions from hospice care upon enrolment”. This quantitative definition allows us to presume that the majority of patients in our study experienced burdensome transfers. In qualitative terms, burdensome transitions may be defined as the presence of a poor physician–patient relationship, which may be caused by either a lack of communication or a lack of trust between physicians and patients [24]. In order to reduce the number of transfers near death, the initial step is the identification of potentially burdensome transitions. Furthermore, this can also prevent the negative health outcomes associated with such transitions (Hanna et al., 2021), for instance, an elevated probability of both inappropriate treatments and medical errors [14]. A study conducted by Schippel et al. [25] indicates that knowledge of the patient’s preferred place of death may also serve to reduce the risk of burdensome transitions. In cases where healthcare personnel were aware of the patient’s preferred place of death, there was a reduction in the number of hospitalisations in the final three months of life [25].

If the decision for transfer is not the patient’s own, this could mean that the patient’s wish for place of death is unfulfilled [25, 26]. Such action could mean that the patient’s opportunity for a good and dignified death is jeopardized. Discussions about a good death and the feeling of experiencing safe care appear in previous research [11, 27–29]. In this context, a good death and feeling of safety can mean trusting the care, having control over symptoms, participating in decision-making, still being able to give something to others, and maintaining autonomy and control over one’s life [29, 30]. This means that patients close to death can change their opinion about the desired place of death on occasions when the home can no longer offer security. Some patients may even sacrifice symptom relief in order to remain at home [31], while others may abandon their desired place of death to make it easier for their caring relatives [11, 31]. At other times, the patient’s relatives experience the caring situation at home as unsafe and, therefore, decide that the patient cannot remain at home [11, 29, 32]. Even if the relatives are aware that it is against the patient’s wish, they rarely regret their decision afterward [29, 32].

Our results also showed that patients in the transferred group experienced a higher degree of symptoms, such as anxiety and dyspnoea than patients in the other group. This is in line with a previous study that showed that cancer patients experiencing these symptoms were more often admitted to hospitals and palliative care units during their last week of life [33]. Furthermore, according to our results, several simultaneous symptoms were more commonly present among patients transferred to inpatient care, indicating that a greater burden of symptoms increases this probability. Identifying and alleviating symptoms can be challenging, especially when patients are so affected by symptoms and illness that they cannot convey their feelings themselves [34]. Additionally, Henson et al. [35] report that patients with advanced cancer often experience an intensification of symptoms as they approach the time of death. Moreover, the study results indicate that this patient cohort requires support through the provision of specialized palliative care [35]. The findings of our study indicate that despite patients receiving specialized palliative care at home, transfers to inpatient care do occur. This implies that healthcare personnel in these contexts would benefit from enhanced training in the assessment and management of complex and multiple symptoms within a home environment. At the same time, there should be an understanding that even patients treated with SPHC can find themselves in acute situations that are not directly linked to the underlying disease. For example, if a patient enrolled in SPHC falls at home and sustains a fracture, there is a legitimate need for transfer to inpatient care. This assumption is corroborated by Pulst et al. [36], who conducted a study on unplanned hospital transfers from nursing homes. Altogether, our study design could not determine why these transfers take place. We call for more studies to determine the underlying causes of transfers to inpatient care. The research needs to address different perspectives, as several different actors are involved in the decisions made in these situations.

As shown by the results of our study, not all patients transferred from SPHC were admitted to a specialized palliative inpatient care unit. This can, to some extent, be explained by the fact that there were significantly more patients with diseases such as cardiovascular disease, lung disease, fractures, and infections who were admitted to non-specialized palliative inpatient care units. Illnesses and conditions of this nature may lead to admission to a specialized ward for the condition requiring treatment rather than the need for specialized palliative inpatient care. However, SRPC does not allow us to identify the reason why a patient has been admitted to a non-specialized palliative inpatient care unit.

We chose to include 221 patients transferred from home to other care units outside a hospital. However, there has been a transfer of these patients, which is why these patients should also be visible in the patient flow. The fact that patients enrolled in SPHC are transferred to special accommodation/nursing and care accommodation/LSS; short-term care facility; or other is noteworthy. This is of interest, and future studies should investigate the reason why patients close to death are transferred to care facilities with a lower level of care.

### Strengths and limitations

To our knowledge, this is the first study to show the extent to which patients near death enrolled in SPHC are being transferred to inpatient care prior to death in Sweden. However, our study has some limitations. The registry’s coverage rate is approximately 55–60% of all deaths [37], but we believe the coverage rate for deaths in the palliative sector is higher, since the majority of hospital wards, palliative care units and home care units report to the SRPC. Furthermore, as already stated, our study does not capture deaths during transfer or in the emergency department.

The quality of the data obtained will always depend on how accurately it was recorded in the registry. For example, the staff at the place-of-death units record the data to the SRPC retrospectively from the time of death; this data should reflect the patient’s last week of life based on information retrieved from the medical record. It seems likely that the information in the SRPC relates only to the period of terminal care at the place of death for patients who were hospitalised for less than one week before death, given the differences between the various medical record systems. Because of this, the staff may not have insight into the patient’s last days and/or do not always have time to familiarize themselves with the patient’s situation, a fact that may call into question the healthcare personnel’s ability to accurately assess the patients’ experiences during this time.

In analysing concurrent symptoms/symptom burden, we have chosen to group together symptoms that may objectively express themselves in similar ways, such as confusion and anxiety, as well as dyspnoea and respiratory secretion. This is because it is healthcare personnel who retrospectively report the possible occurrence of symptoms to the SRPC. In some instances, the reporting of symptoms may be based exclusively on the clinical judgement of the healthcare personnel, which may not fully align with the patient’s actual experience of the symptoms.

A regression model could produce information about risk factors for transfers to hospital. Still, due to the nature of the data, study design and the aim, we chose to present the data on a descriptive level with two group comparisons. The analyses presented in this study are prone to confounders.

Finally, the large volume of data analysed means that even small differences can be statistically significant. Therefore, the results need to be interpreted from the perspective of clinical relevance.

## Conclusions

The results of our study show that patients admitted to SPHC in Sweden are being transferred to inpatient care close to death. A notable proportion of these patients dies within one or two days of admission. Complex symptoms may occur, and not all patients are admitted to specialized palliative inpatient care units. Some common features, such as symptoms and symptom burden, can be observed in the patients transferred. However, further studies are required to identify the causal relationship between these transfers. The study highlights a phenomenon that may be experienced by patients, relatives and healthcare personnel involved as a significant event in a vulnerable situation. A deeper understanding of the underlying causes of these transfers is required to ascertain whether they are compatible with good palliative care and a dignified death.

## Data Availability

The datasets generated and analysed in this study are not publicly available due to Swedish legislation but are available from the corresponding author on reasonable request.

## Abbreviations

SPHC: Specialized palliative home care
SRPC: The Swedish Register of Palliative Care

## References

[1] United Nations. World population prospects 2022: summary of results [UN DESA/POP/2021/TR/NO. 3]. New York: United Nations. Department of Econimic and Social Affairs, Population Division; 2022.

[2] Morin L, Aubry R, Frova L, MacLeod R, Wilson DM, Loucka M, et al. Estimating the need for palliative care at the population level: a cross-national study in 12 countries. Palliat Med. 2017;31(6):526–36. 27683475 10.1177/0269216316671280

[3] World Health Organization. Palliative care - fact sheet. 2023. https://www.who.int/europe/news-room/fact-sheets/item/palliative-care. Accessed 1 June 2023.

[4] International Association for Hospice and Palliative Care (IAHPC). Consensus-based definition of palliative care. 2019. https://hospicecare.com/what-we-do/projects/consensus-based-definition-of-palliative-care/definition/. Accessed 2019.

[5] The National Board of Health and Welfare. Nationellt kunskapsstöd för god palliativ vård i livets slutskede Vägledning, rekommendationer och indikatorer. Stöd för styrning och ledning. 2013–6–4 ed. Västerås: Socialstyrelsen; 2013.

[6] The National Board of Health and Welfare. Palliativ vård – förtydligande och konkretisering av begrepp. 2018–8–6 ed. Socialstyrelsen; 2018. https://www.socialstyrelsen.se/globalassets/sharepoint-dokument/artikelkatalog/ovrigt/2018-8-6.pdf.

[7] Regionala Cancercentrum. Palliativ vård – Nationellt vårdprogram 3.4 ed. Regionala Cancercentrum; 2023. https://kunskapsbanken.cancercentrum.se/globalassets/vara-uppdrag/rehabilitering-palliativ-vard/vardprogram/nationellt-vardprogram-palliativ-vard.pdf.

[8] Cai J, Zhang L, Guerriere D, Fan H, Coyte PC. Where do cancer patients in receipt of home-based palliative care prefer to die and what are the determinants of a preference for a home death? Int J Environ Res Public Health. 2021;18(1):235. 10.3390/ijerph18010235 PMC7796022 33396880

[9] Gomes B, Calanzani N, Gysels M, Hall S, Higginson IJ. Heterogeneity and changes in preferences for dying at home: a systematic review. BMC Palliat Care. 2013;12(1):7. 10.1186/1472-684x-12-7. 23414145 10.1186/1472-684x-12-7 PMC3623898

[10] Nysæter TM, Olsson C, Sandsdalen T, Wilde-Larsson B, Hov R, Larsson M. Preferences for home care to enable home death among adult patients with cancer in late palliative phase – a grounded theory study. BMC Palliat Care. 2022;21(1):49. 10.1186/s12904-022-00939-y. 35410199 10.1186/s12904-022-00939-y PMC9004171

[11] Mertens F, Sercu M, Derycke A, Naert L, Deliens L, Deveugele M, et al. Patients’ experiences of transfers between care settings in palliative care: an interview study. Ann Palliat Med. 2022;11(9):2830–43. 35989649 10.21037/apm-22-146

[12] Wilson DM, Birch S. A scoping review of research to assess the frequency, types, and reasons for end-of-life care setting transitions. Scand J Public Health. 2020;48(4):376–81. 30102574 10.1177/1403494818785042

[13] Salifu Y, Bayuo J. Transfer and transitioning between palliative care settings. Ann Palliat Med. 2022;11(10):3035039–3039. 10.21037/apm-22-1057 36367006

[14] Guo P, Pinto C, Edwards B, Pask S, Firth A, O’Brien S, et al. Experiences of transitioning between settings of care from the perspectives of patients with advanced illness receiving specialist palliative care and their family caregivers: a qualitative interview study. Palliat Med. 2022;36(1):124–34. 34477022 10.1177/02692163211043371 PMC8793309

[15] Pivodic L, Pardon K, Miccinesi G, Alonso TV, Moreels S, Donker GA, et al. Hospitalisations at the end of life in four European countries: a population-based study via epidemiological surveillance networks. J Epidemiol Community Health. 2016;70(5):430–6. 26584859 10.1136/jech-2015-206073

[16] Walsh EG, Wiener JM, Haber S, Bragg A, Freiman M, Ouslander JG. Potentially avoidable hospitalizations of dually eligible Medicare and Medicaid beneficiaries from nursing facility and home-and community-based services waiver programs. J Am Geriatr Soc. 2012;60(5):821–9. 22458363 10.1111/j.1532-5415.2012.03920.x

[17] The Swedish Register of Palliative Care. Årsrapport för Svenska palliativregistret 2021. Svenska palliativregistret; 2023. https://palliativregistret.se/media/xstjwqqa/%C3%A5rsrapport-2021.pdf.

[18] The Swedish Register of Palliative Care. Previous questionnaires used for registering deaths. 2022. https://palliativregistret.se/arkiv/aldre-enkater/. Accessed 2022.

[19] The Swedish Register of Palliative Care. Questionnaire for close family. 2022. https://palliativregistret.se/media/n0jpwlq2/na-rsta-endeenka-t-engelsk.pdf. Accessed 2022.

[20] Corp. I. IBM SPSS Statistics 28.0.0.0 ed. Armonk: IBM Corp; 2021.

[21] Larsdotter C, Nyblom S, Gyllensten H, Furst C-J, Ozanne A, Hedman R, et al. Trends in the place of death in Sweden from 2013 to 2019–disclosing prerequisites for palliative care. Palliat Care Soc Pract. 2024;18:26323524241238230. 38497045 10.1177/26323524241238232 PMC10943753

[22] Singh J, Grov EK, Turzer M, Stensvold A. Hospitalizations and re-hospitalizations at the end-of-life among cancer patients; a retrospective register data study. BMC Palliat Care. 2024;23(1):39. 38350961 10.1186/s12904-024-01370-1 PMC10863145

[23] Christ SM, Huynh M, Schettle M, Ahmadsei M, Blum D, Hertler C, et al. Prevalence and predictors for 72-h mortality after transfer to acute palliative care unit. Support Care Cancer. 2022;30(8):6623–31. 35501514 10.1007/s00520-022-07075-6 PMC9213309

[24] Hanna N, Quach B, Scott M, Qureshi D, Tanuseputro P, Webber C. Operationalizing burdensome transitions among adults at the end of life: a scoping review. J Pain Symptom Manage. 2021;61(6):1261–77. 33096215 10.1016/j.jpainsymman.2020.10.018

[25] Schippel N, Dust G, von Reeken C, Voltz R, Strupp J, Rietz C. Can we determine burdensome transitions in the last year of life based on time of occurrence and frequency? An explanatory mixed-methods study. Palliat Support Care. 2022;20(5):637–45. 36111733 10.1017/S1478951521001395

[26] Marincowitz C, Preston L, Cantrell A, Tonkins M, Sabir L, Mason S. What influences decisions to transfer older care-home residents to the emergency department? A synthesis of qualitative reviews. Age Ageing. 2022;51(11):afac257. 36413591 10.1093/ageing/afac257 PMC9681131

[27] Pedrosa Carrasco AJ, Bezmenov A, Sibelius U, Berthold D. How safe do dying people feel at home? Patients’ perception of safety while receiving specialist community palliative care. Am J Hosp Palliat Med. 2023;40(8):829–36. 10.1177/10499091221140075. 10.1177/10499091221140075 PMC10333965 36396608

[28] Milberg A, Friedrichsen M, Jakobsson M, Nilsson E-C, Niskala B, Olsson M, et al. Patients’ sense of security during palliative care—what are the influencing factors? J Pain Symptom Manage. 2014;48(1):45–55. 10.1016/j.jpainsymman.2013.08.021. 24801659 10.1016/j.jpainsymman.2013.08.021

[29] Rainsford S, Phillips CB, Glasgow NJ, MacLeod RD, Wiles RB. The ‘safe death’: an ethnographic study exploring the perspectives of rural palliative care patients and family caregivers. Palliat Med. 2018;32(10):1575–83. 30229700 10.1177/0269216318800613

[30] Krikorian A, Maldonado C, Pastrana T. Patient’s perspectives on the notion of a good death: a systematic review of the literature. J Pain Symptom Manage. 2020;59(1):152–64. 10.1016/j.jpainsymman.2019.07.033. 31404643 10.1016/j.jpainsymman.2019.07.033

[31] Rainsford S, MacLeod RD, Glasgow NJ. Place of death in rural palliative care: a systematic review. Palliat Med. 2016;30(8):745–63. 26944531 10.1177/0269216316628779

[32] Mertens F, Vanderstichelen S, Deveugele M, Deliens L, Pype P. Family carers’ experiences regarding patient transfers between care settings in palliative care: an interview study. Ann Palliat Med. 2023;12(4):767–82. 37431219 10.21037/apm-23-20

[33] Elmstedt S, Mogensen H, Hallmans D-E, Tavelin B, Lundström S, Lindskog M. Cancer patients hospitalised in the last week of life risk insufficient care quality – a population-based study from the Swedish Register of Palliative Care. Acta Oncol. 2019;58(4):432–8. 10.1080/0284186x.2018.1556802. 30633611 10.1080/0284186x.2018.1556802

[34] Lundin E, Godskesen TE. End-of-life care for people with advanced dementia and pain: a qualitative study in Swedish nursing homes. BMC Nurs. 2021;20(1):48. 10.1186/s12912-021-00566-7. 33743691 10.1186/s12912-021-00566-7 PMC7981921

[35] Henson LA, Maddocks M, Evans C, Davidson M, Hicks S, Higginson IJ. Palliative care and the management of common distressing symptoms in advanced cancer: pain, breathlessness, nausea and vomiting, and fatigue. J Clin Oncol. 2020;38(9):905–14. 32023162 10.1200/JCO.19.00470 PMC7082153

[36] Pulst A, Fassmer AM, Schmiemann G. Unplanned hospital transfers from nursing homes: who is involved in the transfer decision? Results from the HOMERN study. Aging Clin Exp Res. 2021;33:2231–41. 33258074 10.1007/s40520-020-01751-5 PMC8302553

[37] The Swedish Register of Palliative Care. Årsrapport för Svenska palliativregistret 2022. 2024.

[38] Patientdatalag. (2008:355). In: Socialdepartementet, editor. Stockholm: Socialdepartementet; 2008.

